# Genome-wide discovery and characterization of maize long non-coding RNAs

**DOI:** 10.1186/gb-2014-15-2-r40

**Published:** 2014-02-27

**Authors:** Lin Li, Steven R Eichten, Rena Shimizu, Katherine Petsch, Cheng-Ting Yeh, Wei Wu, Antony M Chettoor, Scott A Givan, Rex A Cole, John E Fowler, Matthew M S Evans, Michael J Scanlon, Jianming Yu, Patrick S Schnable, Marja C P Timmermans, Nathan M Springer, Gary J Muehlbauer

**Affiliations:** 1 Department of Agronomy and Plant Genetics, University of Minnesota, Saint Paul, MN 55108, USA; 2 Department of Plant Biology, University of Minnesota, Saint Paul, MN 55108, USA; 3 Department of Plant Biology, Cornell University, Ithaca, NY 14853, USA; 4 Cold Spring Harbor Laboratory, Cold Spring Harbor, NY 11724, USA; 5 Department Agronomy, Iowa State University, Ames, IA 50011, USA; 6 Center for Plant Genomics, Iowa State University, Ames, IA 50011-3650, USA; 7 Department of Plant Biology, Carnegie Institution for Science, Stanford, CA 94305, USA; 8 Informatics Research Core Facility, University of Missouri, Columbia, MO 65211, USA; 9 Department of Botany and Plant Pathology, Oregon State University, Corvallis, OR 97331, USA; 10 Current address: Pioneer Hi-Bred, Johnston, IA 50131, USA

## Abstract

**Background:**

Long non-coding RNAs (lncRNAs) are transcripts that are 200 bp or longer, do not encode proteins, and potentially play important roles in eukaryotic gene regulation. However, the number, characteristics and expression inheritance pattern of lncRNAs in maize are still largely unknown.

**Results:**

By exploiting available public EST databases, maize whole genome sequence annotation and RNA-seq datasets from 30 different experiments, we identified 20,163 putative lncRNAs. Of these lncRNAs, more than 90% are predicted to be the precursors of small RNAs, while 1,704 are considered to be high-confidence lncRNAs. High confidence lncRNAs have an average transcript length of 463 bp and genes encoding them contain fewer exons than annotated genes. By analyzing the expression pattern of these lncRNAs in 13 distinct tissues and 105 maize recombinant inbred lines, we show that more than 50% of the high confidence lncRNAs are expressed in a tissue-specific manner, a result that is supported by epigenetic marks. Intriguingly, the inheritance of lncRNA expression patterns in 105 recombinant inbred lines reveals apparent transgressive segregation, and maize lncRNAs are less affected by *cis-* than by *trans-*genetic factors.

**Conclusions:**

We integrate all available transcriptomic datasets to identify a comprehensive set of maize lncRNAs, provide a unique annotation resource of the maize genome and a genome-wide characterization of maize lncRNAs, and explore the genetic control of their expression using expression quantitative trait locus mapping.

## Background

While the central dogma defines the primary role for RNA as a messenger molecule in the process of gene expression, there is ample evidence for additional functions of RNA molecules. These RNA molecules include small nuclear RNAs (snRNAs), small nucleolar RNAs (snoRNAs; mainly tRNAs and rRNAs), signal recognition particle (7SL/SRP) RNAs, microRNAs (miRNAs), small interfering RNAs (siRNAs), piwi RNAs (piRNAs) and *trans*-acting siRNAs (ta-siRNAs), natural *cis*-acting siRNAs and long noncoding RNAs (lncRNAs). lncRNAs have been arbitrarily defined as non-protein coding RNAs more than 200 bp in length, distinguishing them from short noncoding RNAs such as miRNAs and siRNAs [1,2]. Rather, lncRNAs have been reported to influence the expression of other genes [2]. Based on the anatomical properties of their gene loci, lncRNAs were further grouped into antisense lncRNAs, intronic lncRNAs, overlapping lncRNAs that in part overlap protein-coding genes and intergenic lncRNAs [2]. lncRNAs are usually expressed at low levels, lack conservation among species and often exhibit tissue-specific/cell-specific expression patterns [3,4].

With the advent of genomic sequencing techniques, genome-wide scans for lncRNAs have been conducted via cDNA/EST *in silico* mining [5,6], whole genome tilling array and RNA-seq approaches [7,8] and epigenetic signature-based methods [9,10]. Thousands of lncRNAs have been identified in a number of species. For example, approximately 10,000 human lncRNAs were uncovered by the ENCODE Project [4]. The finding that several hundred human lncRNAs interact with chromatin remodeling complexes suggests that they have functional significance [9]. Indeed, some lncRNAs have been shown to influence human disease, plant development, and other biological processes [10-14].

Although less well characterized than mammalian lncRNAs, plant lncRNAs have defined functional roles. Vernalization in *Arabidopsis* is influenced by lncRNAs *COOLAIR* (an antisense lncRNA) and *COLDAIR* (an intronic lncRNA) [15,16]. *INDUCED BY PHOSPHATE STARVATION1* is a member of the *TPS1/Mt4* gene family that acts as a miR399 target mimic in fine tuning of *PHO2* (encoding an E2 ubiquintin conjugase-related enzyme) expression and phosphate uptake in *Arabidopsis*, tomato and *Medicago truncatula* but does not encode a protein [17,18]. *Enod40* was also identified as a lncRNA involved in nodulation [19,20]. Genome-wide scans for lncRNAs have also been performed in *Arabidopsis thaliana*[21-27], *Medicago truncatula*[28], *Oryza sativa*[29] and *Zea mays*[30]. In maize, an *in silico* bioinformatic pipeline was used on a limited set of full-length cDNA sequences to identify 1,802 lncRNAs, of which 60% are likely to be precursors of small RNAs [30]. Each of the lncRNA surveys in plants has uncovered a substantial number of lncRNAs, which are often expressed at low levels in a tissue-specific manner as in humans and other mammals, and act as natural miRNA target mimics, chromatin modifiers or molecular cargo for protein re-localization [1].

To identify a more comprehensive set of maize lncRNAs, we integrated the information from available public ESTs, maize whole genome sequence annotation, and RNA-seq datasets from 30 different experiments and developmental stages in the reference genotype of maize-B73. In total, 1,704 high-confidence lncRNAs (HC-lncRNAs) and 18,459 pre-lncRNAs (which are likely to be precursors of small RNAs) were identified in this analysis. The expression patterns and potential regulatory roles of these lncRNAs were examined in 30 B73 experiments and at several well-characterized loci. Finally, we explored the regulatory variation of lncRNAs in an RNA-seq dataset of shoot apices from 105 genotypes of the maize intermated B73 × Mo17 recombinant inbred line (IBM-RIL) population [31] to map the genetic factors underlying the expression variation of lncRNAs. These expression quantitative trait locus (eQTL) mapping results enhance our understanding of the inheritance of lncRNA expression in plants.

## Results

### Genome-wide identification of lncRNAs in maize

**Figure 1:**
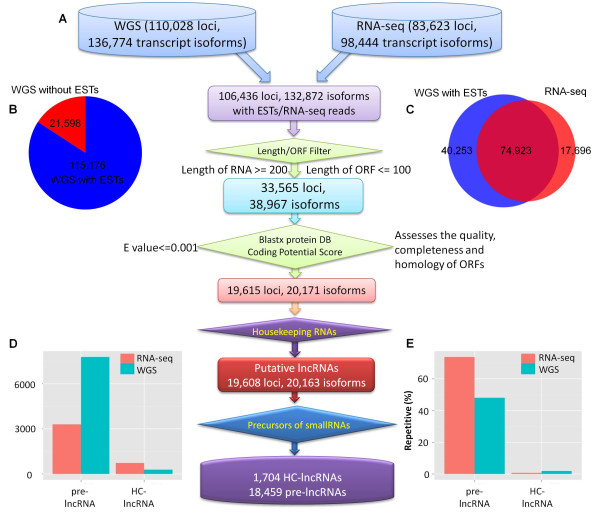
**Informatics pipeline for the identification of maize lncRNAs. (A)** Schematic diagram of the informatics pipeline. **(B)** The proportion of WGS transcripts with/without EST support. **(C)** Venn diagram showing the numbers of transcripts detected by the WGS, RNA-seq assemblies or by both assemblies. **(D)** The number of HC-lncRNAs and pre-lncRNAs derived from RNA-seq and WGS, respectively. **(E)** The proportion of transcripts from the WGS and RNA-seq with sequence similarity to maize repetitive elements. DB, DataBase; EST, expressed sequence tag; HC-lncRNAs, high confidence lncRNAs; ORF, open reading frame; WGS, working gene set.

We sought to identify a relatively comprehensive set of maize lncRNAs. To achieve this, it is important to remove potential pseudogenes that have acquired nonsense or missense mutations as well as non-coding RNA precursors that will give rise to known classes of RNAs such as tRNAs, rRNA, and snRNAs. A comprehensive set of transcripts for the reference genotype B73 was developed by combining data from two sources: the maize working gene set transcripts [32]; and *de novo* transcript assemblies from RNA-seq datasets from 30 different experiments (Figure 1A). There are 110,028 loci (136,774 transcript isoforms) in the working gene set (WGS) of the maize genome annotation [33]. This set of genes consists of both computational predictions of genes as well as EST collections from a variety of tissues. Many analyses in maize utilize the 39,656 genes in the filtered gene set (FGS), a subset of the WGS that was selected based upon sequence similarity to other species and the existence of putative full-length coding sequences [32]. However, the WGS may include lncRNAs [30]. We also developed a set of transcript assemblies based upon 806 million uniquely mapped reads from 30 different experiments of the reference genotype-B73 [34-39]. These sequences were used to perform *de novo* transcript assembly with Cufflinks [40] and resulted in 83,623 expressed loci with 98,444 transcript isoforms, of which 16,759 loci and 17,696 transcript isoforms are not present in the WGS. The 110,028 loci (136,774 transcript isoforms) from the WGS and 83,623 loci (98,444 transcript isoforms) from the *de novo* transcript assemblies were combined to generate a non-redundant set of 126,787 transcribed loci (154,470 transcript isoforms) (Figure 1B,C).

This comprehensive set of transcribed sequences from B73 was analyzed to identify putative lncRNAs. There are 33,565 loci (38,967 transcript isoforms) that are at least 200 bp in length and do not encode an ORF of more than 100 amino acids. These sequences were filtered by comparing with the Swiss-Protein database to eliminate transcripts that contain sequence similarity (E-value ≤0.001) to known protein domains. Further filtering was performed using the Coding Potential Calculator [41], which assesses the quality, completeness and sequence similarity of potential ORFs to proteins in the NCBI protein database. After applying these criteria, we identified 19,608 loci (20,163 transcript isoforms; Additional file 1) that encode transcripts of >200 bp but that have little evidence for coding potential, and that were considered as putative lncRNAs. These include 12,431 loci (12,647 isoforms) from the WGS and 7,177 loci (7,515 isoforms) from the *de novo* transcript assemblies. This set of putative lncRNAs also includes 1,580 sequences previously identified by Boerner and McGinnis [30].

**Figure 2:**
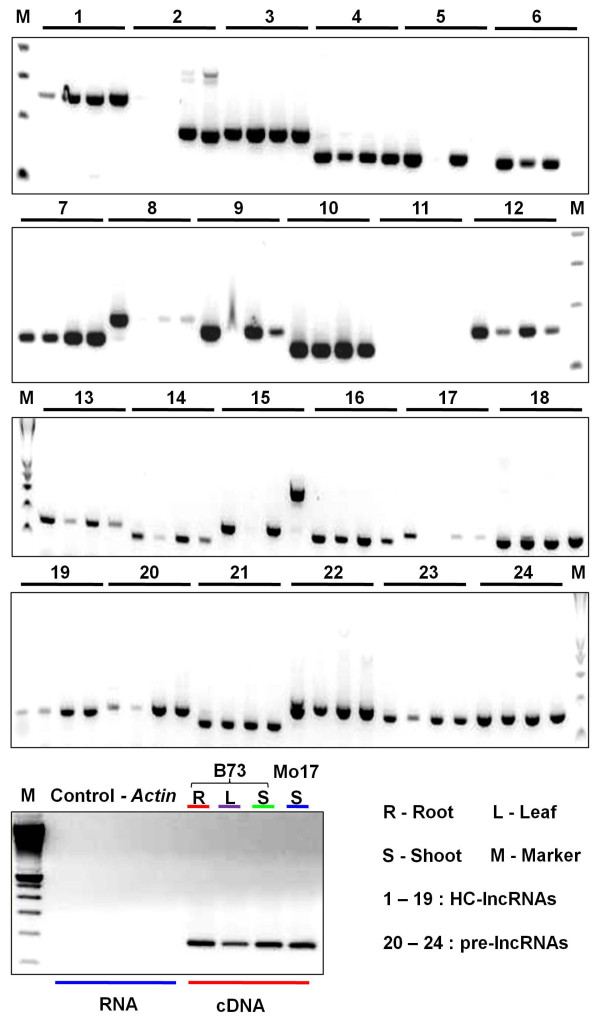
**RT-PCR validation of putative lncRNAs in root, leaf and shoot of 2-week seedlings of maize inbreds (B73 and Mo17).** Twenty-four putative lncRNAs, including 19 HC-lncRNAs and 5 pre-lncRNAs, that exhibit expression in seedling tissue were selected for RT-PCR validation. Each primer set was used to perform RT-PCR on four RNA samples, including (1) B73 root, (2) B73 leaf, (3) B73 shoot, and (4) Mo17 shoot isolated from 2-week-old seedlings. Actin was used as a control to show amplification of cDNA samples but no amplification of untreated RNA samples. The marker is a 100 bp DNA ladder from Invitrogen.

These 20,163 putative lncRNAs may contain precursors to small RNA molecules, such as miRNAs, short hairpin RNAs (shRNAs) and siRNAs [30]. The putative lncRNAs were compared to a comprehensive set of small RNAs from different tissues and small RNA related mutants. More than 90% (18,459) of the putative lncRNAs have sequence similarity with small RNAs and were classified as pre-lncRNAs (Additional file 2; Materials and methods). A set of 1,704 lncRNAs that do not have sequence similarity to known classes of noncoding RNAs were defined as HC-lncRNAs (Additional file 3). These 1,704 HC-lncRNAs include 479 sequences from the WGS and 1,225 sequences from the *de novo* transcript assemblies (Figure 1D). The HC-lncRNAs also contain 201 (35%) of the 572 HC-lncRNAs previously identified by Boerner and McGinnis [30]. RT-PCR was used to validate the expression and sequence for 24 lncRNAs (Figure 2). The 24 putative lncRNAs selected for validation include 18 that were present in the working gene set from the maize genome project [32] and 6 that are novel transcripts from our assembly of RNA-seq data. RT-PCR was performed for root, leaf and shoot tissue of 2-week old B73 seedlings and the expected products were recovered for 23 of the 24 lncRNAs tested. In some cases, there was evidence for tissue-specific expression while many of the lncRNAs were detected in all three tissues. These RT-PCR bands and specific expression were largely consistent (90/96) with the RNA-seq data. For example, lncRNA (*GRMZM2G549431_T01*) was not detected by both RNA-seq and RT-PCR in the leaf sample. Two of the lncRNAs (*GRMZM2G010274_T01* and *GRMZM2G518002_T01*) showed additional isoforms in some of the tissues that may reflect tissue-specific splicing variants. RT-PCR products from 10 lncRNAs were sequenced and all 10 exhibited the appropriate sequence. We proceeded to analyze characteristics, diversity and inheritance patterns of these maize HC-lncRNAs.

### Characterization of maize lncRNAs

**Figure 3:**
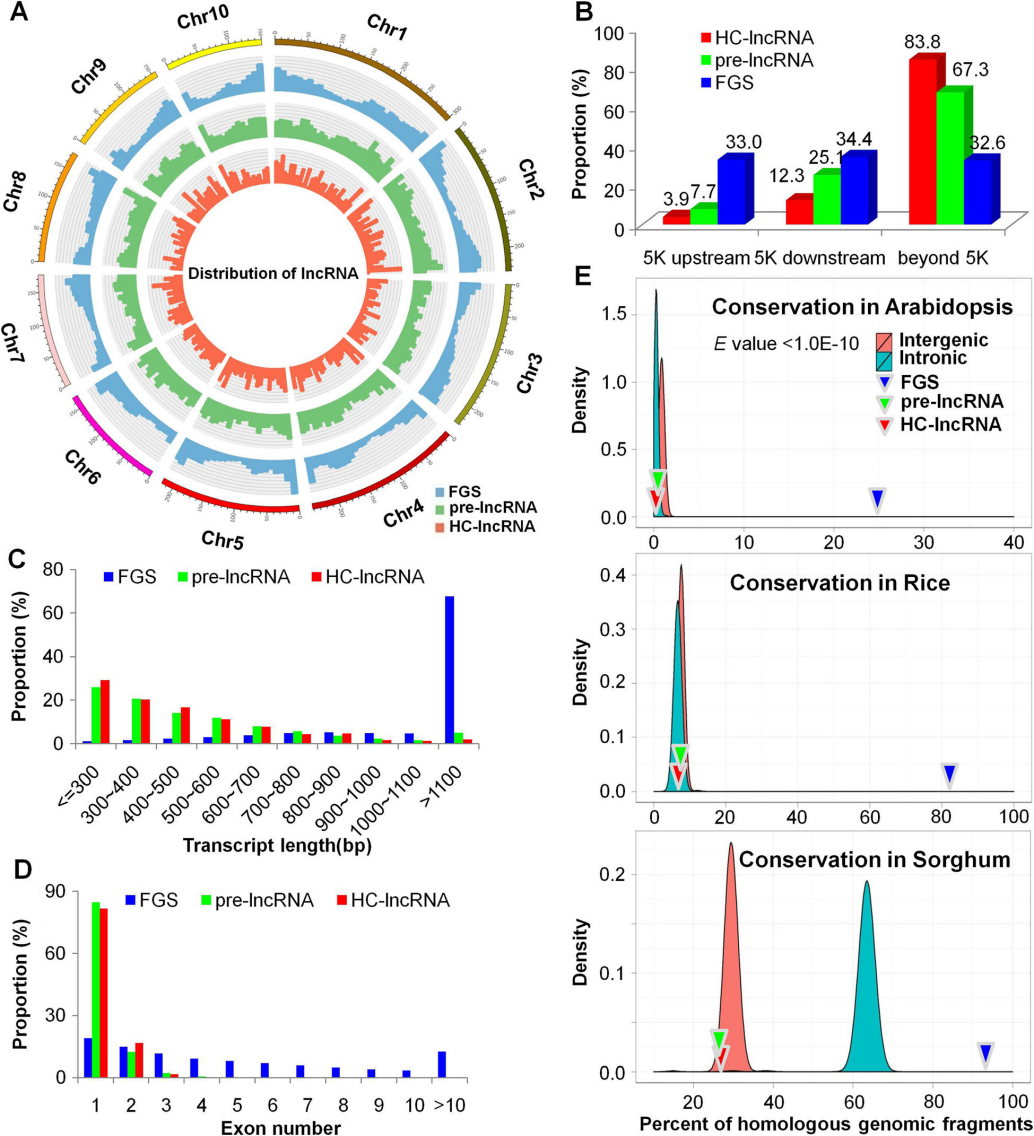
**Characteristics of maize lncRNAs. (A)** Distribution of lncRNAs along each chromosome. The abundance of HC-lncRNAs, pre-lncRNAs and FGS genes in physical bins of 10 Mb for each chromosome (generated using Circos). **(B)** Proportion of HC-lncRNAs and pre-lncRNAs that are located within 5 kb (upstream or downstream) or further than 5 kb from the nearest FGS gene. The proportion of FGS genes located within close proximity to other FGS genes is used as a control. **(C)** Lengths of HC-lncRNAs, pre-lncRNAs and FGS transcripts. **(D)** Numbers of exons in HC-lncRNAs, pre-lncRNAs and FGS. **(E)** Percentage of maize HC-lncRNAs and FGS transcripts that are conserved in the *Arabidopsis*, rice and sorghum genomes compared with the sequence conservation of intergenic or intronic fragments among species. Sequences were repetitive-sequence masked and aligned to the genomes of *Arabidopsis*, rice and sorghum with the significant cutoff E value <1.0E-10.

A substantial number (74%) of the pre-lncRNAs have sequence similarity to repetitive sequences of maize (Figure 1E). In contrast, the majority (98%) of the HC-lncRNAs do not contain maize repetitive sequences. Taken together, over 68% (13,811) of 20,163 maize putative lncRNAs are repetitive sequences (or transposons), which is similar to the proportion of lncRNAs in mammals [42]. While the pericentromeric regions of most maize chromosomes have lower gene densities than chromosome ‘arms’ [32], maize lncRNAs are more evenly distributed across chromosomes (Figure 3A). The HC-lncRNAs were characterized according to the locations relative to the nearest protein-coding genes. The majority of lncRNAs (93%) are located in intergenic regions and only 7% of the lncRNAs overlap with gene sequences. Among the intergenic HC-lncRNAs, 66 (3.9%) and 209 (12.3%) are located within 5 kb upstream and downstream of genes, respectively (Figure 3B). The remaining 83.8% of intergenic lncRNAs are at least 5 kb away from the nearest gene. This proportion (83.8%) is significantly (*P* = 8.1E-09) higher than the proportion of FGS genes located at least 5 kb from other FGS genes (32.6%). The majority of the lncRNAs are relatively short with very few (3%) greater than 1 kb in length (Figure 3C). Most (81%) of the lncRNAs consist of a single exon (Figure 3D). While we could not directly distinguish the transcript orientation using the non-strand-specific RNA-seq, transcript orientations could be determined using the intron splicing ‘GT-AG rule’ for those HC-lncRNA genes that contain an intron. Of the 323 lncRNAs that could be oriented based on the GT-AG intron splice sites, 23 (7%) consist of antisense transcripts.

The lncRNA sequences were compared with genomic sequences from *Arabidopsis*, rice and sorghum to determine the portion of lncRNAs that had similarity (BLASTN E < 1.0E-10) to these species (Figure 3E). As expected, the conservation of lncRNAs is substantially lower than that of protein coding genes in comparisons with all three species. Permutations of random samplings of intergenic or intronic DNA were used to assess whether lncRNAs exhibit the same levels of conservation for these sequences among species. The lncRNAs have sequence similarity at the same rate as observed for intergenic sequences in all three cross-species comparisons. The maize lncRNAs exhibit the same level of conservation in *Arabidopsis* and rice as intronic sequences (*P* > 0.05) but they are significantly less conserved in sorghum (*P* < 0.01) than are randomly selected repeat-masked intronic sequences with similar length distribution to lncRNAs (Figure 3E).

The level of DNA methylation within and surrounding lncRNA genes was compared with that of the FGS genes in the reference genotype B73 (Additional file 4) [43]. Similar levels of DNA methylation are observed in regions 1 kb upstream and downstream of lncRNAs and FGS genes. For both the lncRNAs and FGS genes the level of DNA methylation is reduced near the transcription start and stop sites. FGS genes show substantial levels of gene body methylation in CG and CHG contexts while the gene bodies of lncRNAs do not. Gene body methylation is often associated with genes with moderate to high levels of constitutive expression [44] and the lack of gene body methylation for lncRNAs may reflect lower or more variable expression for these genes. The CHH DNA methylation level is quite low for both FGS and lncRNA sequences.

### Variation in lncRNA expression among tissues

**Figure 4:**
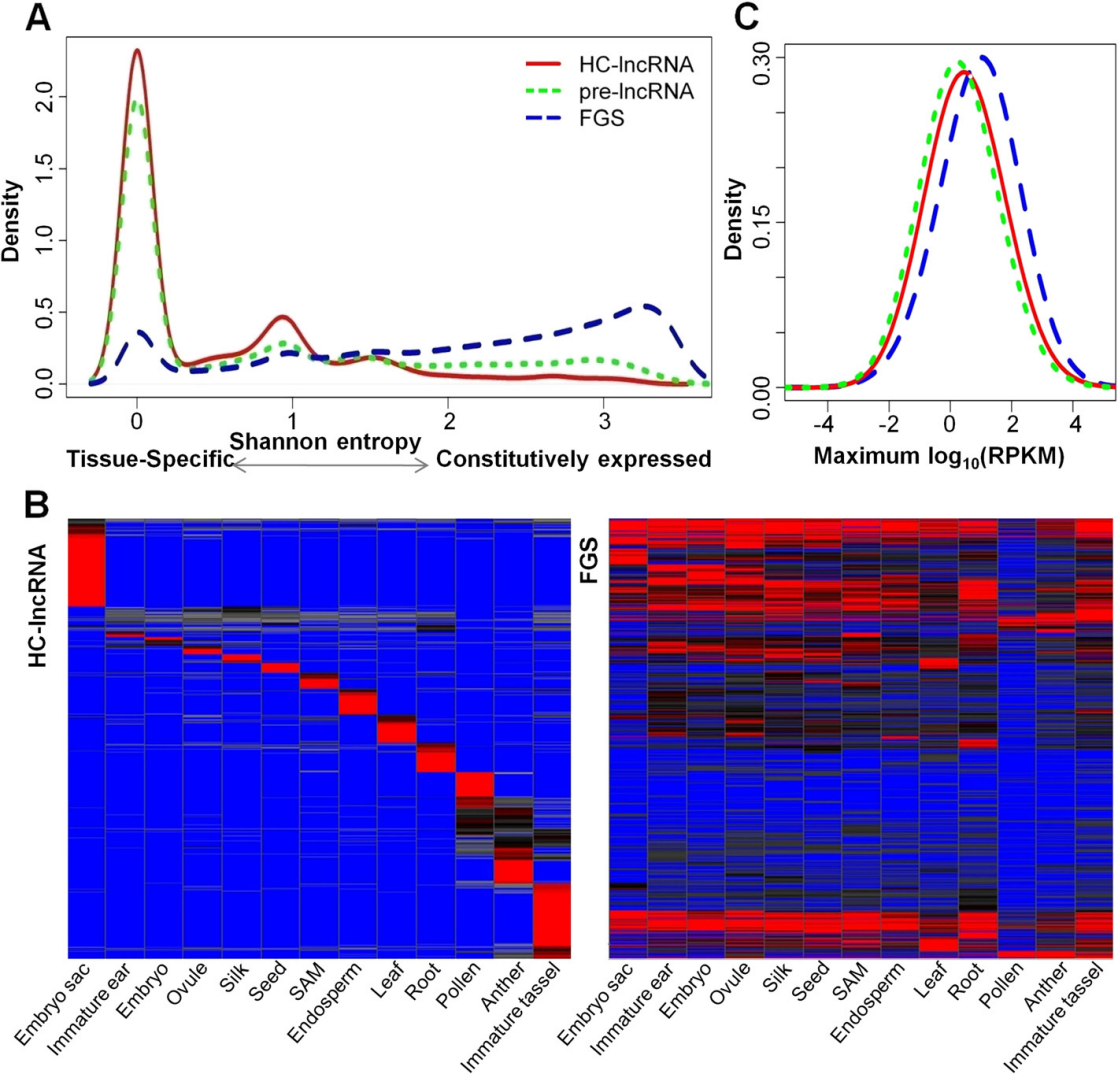
**Tissue-specific expression and expression levels of lncRNAs. (A)** Density plot of Shannon entropy of pre- and HC-lncRNAs, and FGS transcripts. The Shannon entropy has units of bits and ranges from zero for genes expressed in a single tissue to log2(Number of tissues) for genes expressed uniformly in all tissues considered. **(B)** Hierarchical clustering (Ward’s method) of expression for the HC-lncRNAs and FGS genes that were expressed in at least one tissue suggests that tissue-specific expression for lncRNAs is more common than that of FGS genes. Per-gene normalization was applied to allow for visualization of relative expression in different tissues for all genes. Red indicates high expression level, blue low expression, and black intermediate expression. SAM, shoot apical meristem. **(C)** Density plot of maximum expression levels of pre-lncRNAs (green), HC-lncRNAs (red), and FGS (blue) across 13 distinct tissues of B73.

The tissue-specificity of lncRNA expression was explored using the RNA-seq data from 30 different samples of B73 that represent 13 distinct tissue types. The Shannon entropy, which ranges from zero for genes expressed in a single tissue to log2(Number of tissues) for genes expressed uniformly in all tissues considered, was employed to measure the tissue-specificity of lncRNA expression [45]. Many (54%) of the lncRNAs were only detected in one of the tissues (with at least four RNA-seq reads detected) and 10% of the lncRNAs were detected in five or more tissues (Figure 4A,B). In contrast, only 8% of FGS genes were detected in only one tissue and 74% of FGS genes were detected in five or more tissues using the same expression criteria (Figure 4A,B). Interestingly, the male reproductive tissues (immature tassel, anther, and pollen) and embryo sac had more examples of lncRNA expression than other tissues (Figure 4B). An analysis of the maximum expression level (reads per kilobase per million reads (RPKM)) for all 13 tissues provided evidence that FGS genes tend to have higher expression than lncRNAs (Figure 4C). However, 20% of the lncRNAs had an expression of >5 RPKM in at least one tissue, indicating that many of these sequences do show at least moderate expression levels in some tissues. In any one tissue, a higher proportion of FGS genes were expressed relative to HC-lncRNAs and expressed FGS genes had significantly higher expression levels than expressed HC-lncRNAs. This tissue-specific expression for many of the lncRNAs suggests that the expression of these sequences is biologically controlled rather than simply reflecting ‘transcriptional noise’.

H3K27me3 is a facultative heterochromatin mark that is often associated with tissue-specific regulation of gene expression [46]. The levels of H3K27me3 (trimethylation of histone H3 lysine 27) for lncRNAs were assessed in five different tissues [46]. There are differences in the relative abundance of H3K27me3 over lncRNAs in different tissues of maize (Figure S2A in Additional file 5). The tissue with the lowest average level of H3K27me3, immature tassel, also exhibits expression for more of the lncRNAs than the other tissues, suggesting that H3K27me3 may be involved in regulating tissue-specific expression for lncRNAs. To assess the correlation between expression and H3K27me3 for the lncRNAs, H3K27me3 levels were contrasted for the lncRNAs that are expressed or silent in each of the tissues for which H3K27me3 profiles were available for analysis (Figure S2B in Additional file 5). In each tissue, genes were classified as not expressed (RPKM = 0) or expressed (RPKM >1). In general, lncRNAs that are expressed tend to have lower levels (*P* < 0.001) of H3K27me3 while the lncRNAs silenced in any one tissue often have elevated H3K27me3 (Figure S2B in Additional file 5). The presence of H3K27me3 at silenced lncRNAs provides evidence for targeted regulation of the expression of these lncRNAs similar to what is observed at maize genes.

### HC-lncRNAs inheritance pattern in the maize IBM-RIL population

**Figure 5:**
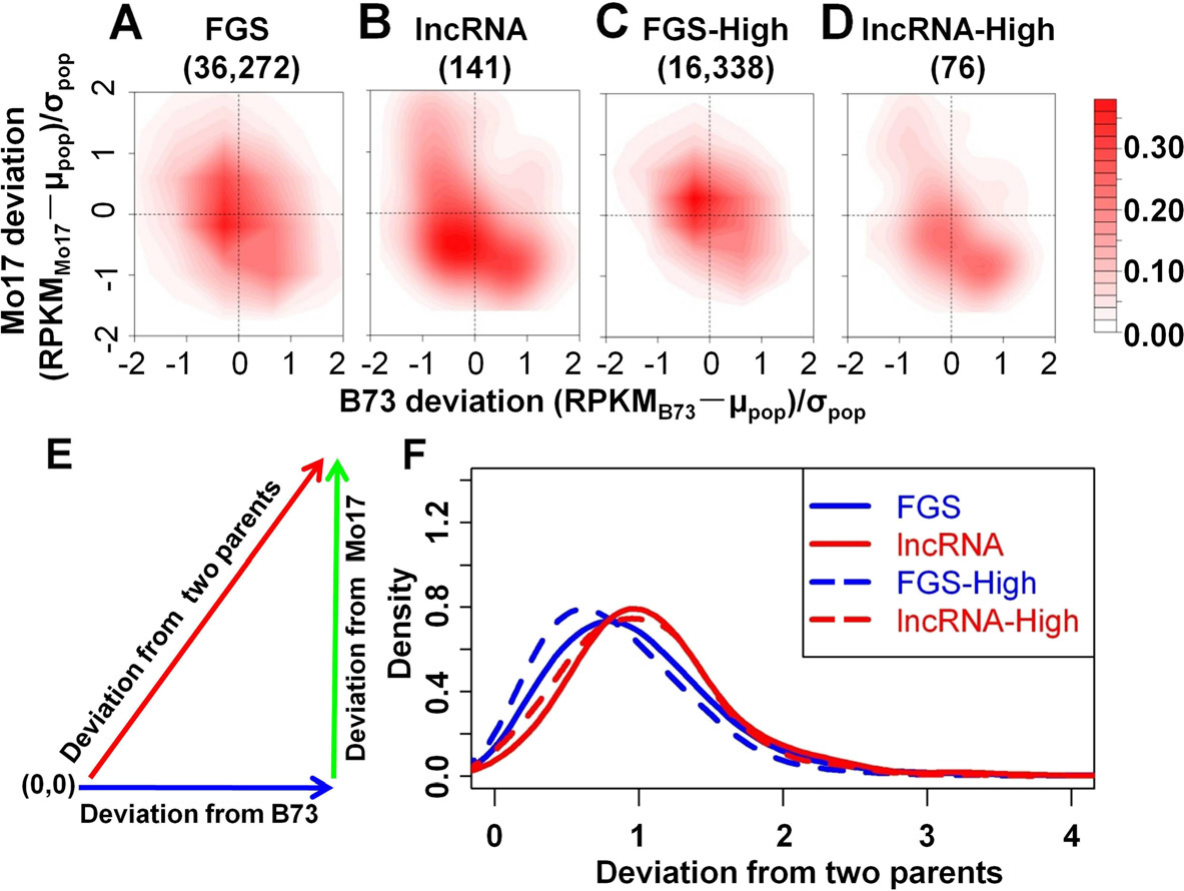
**Inheritance pattern of lncRNAs and FGS genes in 105 maize IBM RILs. (A-D)** Two-dimensional kernel density estimation of gene expression patterns in RILs compared with the two parents for FGS genes and lncRNAs. The x-axis and y-axis represent the expression-level deviation in 105 RILs to the parents B73 and Mo17, respectively. FGS-High, FGS genes with expression level ≥10 RPKM; lncRNA-High, lncRNAs with expression level ≥10 RPKM. **(E)** Schematic diagram of the expression-level deviation in progeny from the two parents. **(F)** Distribution of expression-level deviations of the FGS and lncRNAs in 105 maize IBM RILs from their two parents. (0,0) means the expression levels of transcripts in 105 RILs are similar to the levels of the two parents.

The expression levels of HC-lncRNAs in shoot apices of 105 maize IBM-RILs [31] were compared with the expression levels in the parental lines for the 141 HC-lncRNAs that have detectable expression (at least 4 reads/RIL) in more than 40% of the RILs. The expression patterns of these 141 HC-lncRNAs were compared with those of genes in the FGS. The analysis of expression levels in shoot apices of 105 IBM RILs provides evidence for higher levels of transgressive variation in expression levels of HC-lncRNAs than in FGS genes. The difference in expression for the RILs relative to B73 or Mo17 was compared by calculating (Exp_parents_ - μ_progeny_)/σ_progeny_, which is expected to be centered at zero if the RILs generally have expression levels similar to the parents. In general, the HC-lncRNAs tend to be expressed in the RILs at levels similar to their parents but they have larger variation relative to the parents than observed for FGS genes (Figure 5A,B). This larger variation for HC-lncRNAs than FGS genes may reflect the fact that most HC-lncRNAs have quite low expression levels. However, a targeted analysis of HC-lncRNAs and FGS genes with high expression levels (RPKM >10) revealed that even highly expressed HC-lncRNAs have larger expression variation than FGS genes (Figure 5C,D). The deviation of expression levels from that of the two parents was calculated as a vector (Figure 5E) and shows evidence for higher deviation for HC-lncRNAs than for the FGS genes (*P* = 2.15E-20) (Figure 5F). This difference between HC-lncRNAs and FGS genes is observed for highly expressed transcripts but is not detected in transcripts with differential expression between the parents.

### Genetic dissection of expression-level variation of HC-lncRNAs by eQTL mapping

**Figure 6:**
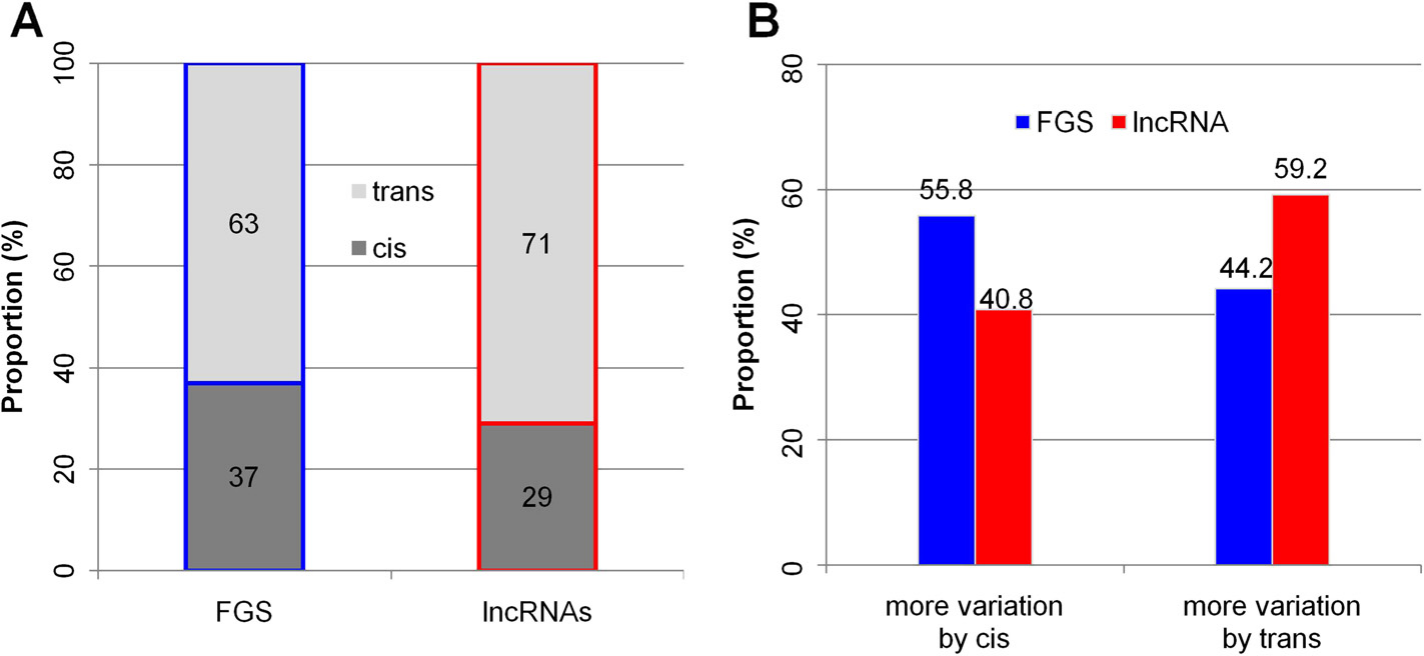
**eQTL mapping of lncRNA expression. (A)** The proportion of *cis*- and *trans*-eQTLs identified for the expression of lncRNAs and FGS genes. **(B)** The proportion of lncRNA expression by *cis*-eQTL with dominant effect and *trans*-eQTLs with dominant effect.

The expression data from the 105 IBM RILs was used to map the regulatory regions of HC-lncRNA expression. eQTL mapping was conducted for 74 HC-lncRNAs detected in at least 80% of maize RILs using the expression levels in the 105 RILs as expression traits and a set of 7,865 high quality SNP markers [31]. A total of 72 eQTLs (α = 0.05) with a threshold logarithm of odds (LOD) ≥4.17 were identified for 49 HC-lncRNAs. The 72 eQTLs include 21 (29%) *cis*-eQTLs and 51 (71%) *trans*-eQTLs (Figure 6A; Additional file 6), of which the proportion of *trans*- versus *cis*-eQTLs is slightly higher (*P* = 3.21E-03) than that observed for FGS genes [31]. Each HC-lncRNA or FGS gene was classified according to whether a higher proportion of expression variation was explained by *cis*- or *trans*-eQTL (Figure 6B). The HC-lncRNAs were more likely to have a major *trans*-acting eQTL than the FGS genes (Figure 6B). Previous eQTL studies in animals and plants have revealed that many loci influenced by multiple *trans*-eQTL have quite high levels of expression variation in segregating offspring, presumably due to the potential for segregation of multiple eQTL with different directional effects that result in transgressive segregation [47]. This could explain why we observe higher levels of transgressive variation for lncRNAs as they are enriched for regulation by *trans*-eQTL relative to FGS genes (Figure 6A). The increased contribution of *trans*-acting regulation to expression variation for HC-lncRNAs is consistent with the observation of higher levels of transgressive segregation for HC-lncRNA expression relative to FGS gene expression.

We also dissected the genetic factors underlying the expression variation of 67 HC-lncRNAs, which were expressed in more than 40% but less than 80% of the RILs, as these may represent HC-lncRNAs that are expressed from one haplotype but not the other. The eQTL mapping for these 67 HC-lncRNAs identified 72 eQTLs that influenced expression of 51 of these HC-lncRNAs (Additional file 7). These HC-lncRNAs are enriched for having pre-dominant effects of *cis-*eQTLs (72.5%) compared with the HC-lncRNAs that are expressed in over 80% of the RILs (40.8%).

Furthermore, 460 HC-lncRNAs were expressed (with at least 4 RNA-seq reads detected) in less than 40% of the RILs. Most (80%) of these HC-lncRNAs were expressed at very low levels (the population mean is less than 5 RPKM); while only 94 HC-lncRNAs were detected with moderate expression levels (Additional file 8). Of these moderately expressed HC-lncRNAs, only six were detected in more than 10% but less than 40% of the 105 RILs. In total, 88 out of 94 moderately expressed HC-lncRNAs were detected in only one of the 105 RILs. Taken together, these results indicate that complex regulatory mechanisms may underlie HC-lncRNA expression variation.

### Potential functional roles for maize lncRNAs

**Figure 7:**
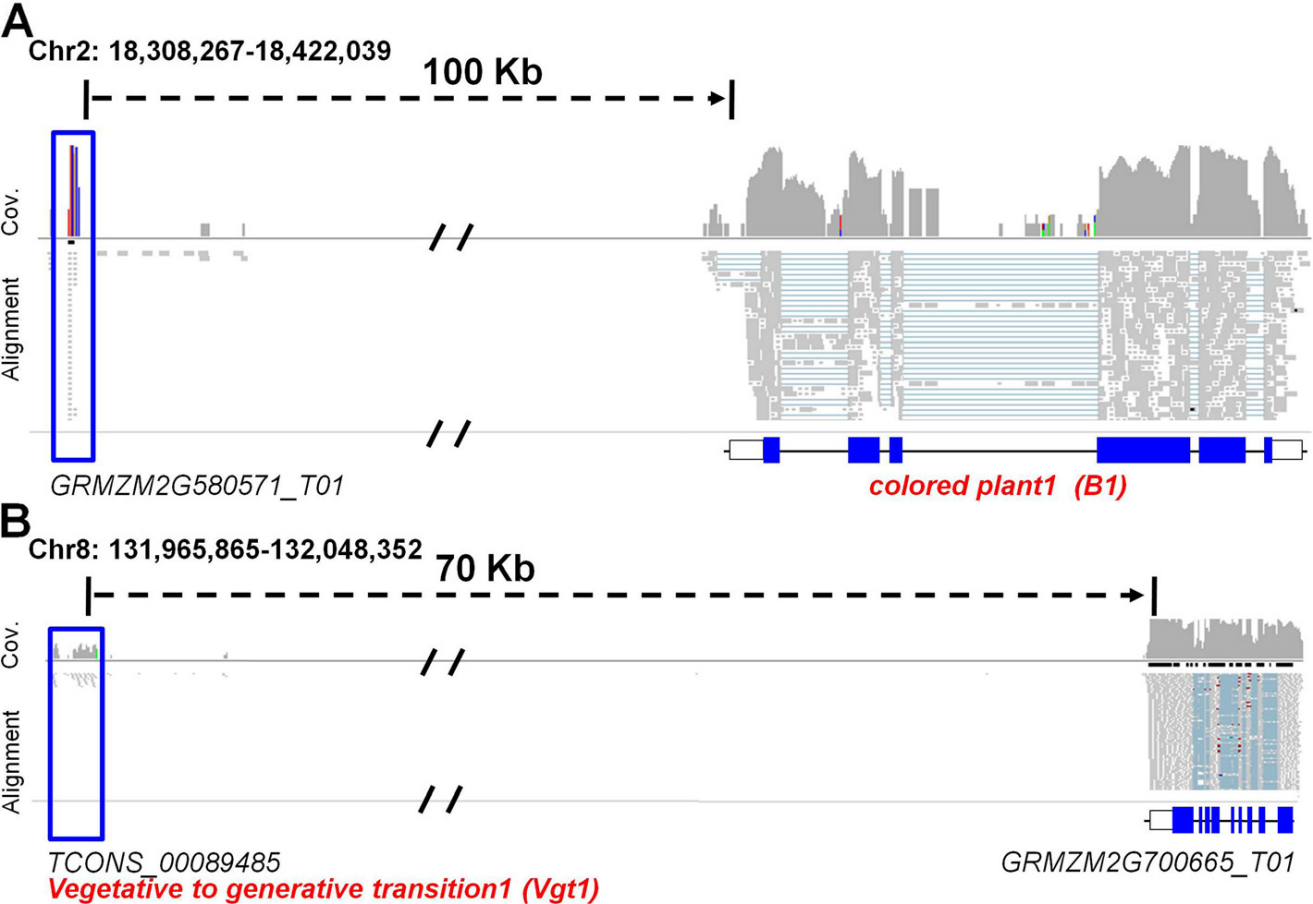
**lncRNAs with potential functions. (A,B)** The structure of lncRNAs at the locus *colored plant1* (*B1*) **(A)**, and *Vegetative to generative transition 1* (*Vgt1*) **(B)**, and their read coverage by RNA-seq on 30 B73 experiments. Each figure has three separate panels showing the RNA-Seq read coverage, read alignment of bulked RNA-Seq data from 30 B73 experiments and gene model from top to bottom. The blue box linking three panels of each figure highlights the lncRNA regions uncovered in our study.

There are relatively few functionally characterized lncRNAs in maize. A careful analysis of the regulation of the *B1* locus in maize identified a region located more than 100 kb upstream of the coding sequence [48] that is important for regulation and paramutation of *B1* expression. There is evidence for expression of a HC-lncRNA from this region [49] that may play a role in paramutation [50,51]. Similarly, we identified a HC-lncRNA (*GRMZM2G580571_T01*) in the regulatory region of *B1*, which was previously identified as required for *B’* paramutation (Figure 7A). There are several other examples of maize genes with long-distance regulatory elements. The map-based cloning of a major flowering time QTL, *Vegetative to generative transition1* (*Vgt1*), identified a conserved non-coding region located 70 kb upstream of the *ZmRap2* (*GRMZM2G700665*) gene, which can influence flowering time [52]. We found a HC-lncRNA (*TCONS_00089485*) that is expressed from the *Vgt1* regulatory region (Figure 7B). This lncRNA is detected in embryo sac and ovule tissues where *ZmRap2* is not detected, while *ZmRap2* is expressed in other tissues where this HC-lncRNA is not detected, suggesting the potential for antagonistic expression of this HC-lncRNA and the nearby coding sequence. The cloning of a major domestication QTL in maize identified the *teosinte branched1* (*tb1*) gene [53]. Further analyses provided evidence for the importance of a distant enhancer located approximately 40 kb upstream of the coding sequence [54] that may be influenced by a transposon insertion [55]. We also identified a pre-lncRNA (*TCONS_00010027*) derived from this genomic region in our study. This pre-lncRNA (*TCONS_00010027*) has sequence similarity with small RNAs and thus may be chopped into pieces and function as a small RNA. Because lncRNAs showed strong tissue-specific expression patterns and relatively low expression levels, and none of these three lnRNAs were detected in the tissue used for the eQTL analysis with 105 maize RILs, we could not conduct eQTL mapping for these lncRNAs. The finding that lncRNAs were detected from distant regulatory regions in all three of these examples suggests that a number of the distant regulatory regions for these maize genes may potentially involve lncRNAs. The shoot apical meristem (SAM), from which all aboveground tissues of plants are derived, is critical to plant morphology and development [56]. While SAM initiation and development is characterized by distinct transcriptional variation [57], we also identified a subset of putative lncRNAs exhibiting distinct expression variation during different stages of SAM ontogeny (Additional file 9). Further research will be necessary to elucidate the functional roles of maize lncRNAs.

## Discussion

The advent of high-resolution tiling arrays, the emergence of new technologies in the field of RNA-seq and large-scale chromatin immunoprecipitation experiments followed by next generation sequencing (ChIP-Seq), as well as cDNA-library sequencing and serial analysis of gene expression (SAGE), have allowed the research community to quantitatively discriminate most of the cellular transcripts [58]. Each technical advance in examining the eukaryotic transcriptome has revealed the increasing complexity of eukaryotic genome expression [59]. One such complexity is the existence of non-protein coding genes, including short non-protein coding genes (such as small interfering RNAs and miRNAs) and long non-protein coding genes. The short noncoding RNAs are relatively well characterized and their importance in transcriptional and posttranscriptional regulation of expression of other genes is well understood [60]. In contrast, lncRNAs have not been as comprehensively identified or studied in many plant species.

Our analysis generated a relatively robust list of potential lncRNAs for maize. This set of lncRNAs will likely be useful for functional genomics research or the analysis of potential functional differences among maize varieties. The lncRNAs detected in this analysis were identified from analysis of RNA-seq data from a diverse set of tissues and the current WGS annotation. In total, more than 20,000 putative lncRNAs, including 1,704 HC-lncRNAs and 18,459 pre-lncRNAs, which are likely precursors of small RNAs, were identified. We have provided GTF files as supplemental tables (Additional files 2 and 3) to enable the use and display of these lncRNAs by other researchers. Our study sheds light on the features and expression inheritance patterns of lncRNAs in maize, but also complements the reference genome annotation of maize, which might further aid the functional gene cloning and trigger more comprehensive studies on gene regulation in plants.

Despite our use of >1 billion RNA-seq reads, it is worth noting that we only detected expression for approximately 80% of the maize FGS and approximately 50% of the lncRNAs (the other half are from WGS annotations). This may indicate that a number of additional lncRNAs with tissue- or environment-specific expression have not been detected. It is worth noting that we applied an RPKM cutoff for identifying expressed lncRNAs and that most lncRNAs were expressed at relatively low levels. While caution is required when quantifying the expression levels of genes with low RNA-seq coverage [61], focusing the analysis on lncRNAs with moderate expression may result in loss of lncRNAs with low expression. There are several other potential limitations to our list of lncRNAs. Most of the WGS, EST/cDNAs, and RNA-seq data were obtained after the reverse transcription with polyA primers, which selected for polyadenylated transcripts, and it is possible that some lncRNAs lack poly-adenylation. We have also employed relatively strict criteria by requiring that the putative lncRNAs lack the ability to encode peptides of more than 100 amino acids or only have a weak coding potential. However, there are examples of previously characterized lncRNAs from other species that have the potential to encode peptides >100 amino acids, such as *HOTAIR* with 106 amino acids [62], *XIST* with 136 amino acids [63] and *KCNQ1OT* with 289 amino acids [64]. These examples are not thought to function as proteins but would not have met our relatively strict criteria for definition as lncRNAs. Although we have identified more than 20,000 lncRNAs, it is likely that additional maize lncRNAs exist and will be discovered through analysis of additional tissues and genotypes or refinement of bioinformatics methods for characterizing lncRNAs.

## Conclusions

As previous studies have suggested [1-28], a substantial number of lncRNAs exist in mammals and plants, and play important functional roles in human disease, plant development, and other biological processes. In this study, we integrated available transcriptome datasets in maize to identify maize lncRNAs. More than 20,000 lncRNAs were uncovered in the maize reference genome B73, of which 1,704 were considered HC-lncRNAs. These HC-lncRNAs showed similar methylation levels as protein coding genes; however, they were more likely to exhibit tissue-specific expression patterns, which were also supported by epigenetic marks. eQTL mapping of the HC-lncRNAs showed that *trans*-eQTL contribute more to the expression-level variation of lncRNAs. Finally, we identified lncRNAs that were derived from regulatory regions controlling *Tb1*, *Vgt1*, and *B1*, which are key genes of developmental and agronomic importance in maize. We present the first comprehensive annotation of lncRNAs in maize, which opens the door for future functional genomics studies and regulatory expression research. Our findings constitute a valuable genomic resource for the identification of lncRNAs underlying plant development and agronomic traits. We also identified potential genetic mechanisms that control expression variation of lncRNAs in plant genomes.

## Materials and methods

### Datasets used for lncRNA identification

Transcribed sequences from the maize reference inbred line B73 were collected from the Sequence Read Archive [65] and GenBank [66]. Data available in the Sequence Read Archive from the maize inbred line B73 included 30 RNA-seq experiments from 13 distinct tissues (leaf, immature ear, immature tassel, seed, endosperm, embryo, embryo sac, anther, ovule, pollen, silk, and root and shoot apical meristem) encompassing a total of 1.168 billion reads with read lengths ranging from 35 to 110 nucleotides (Additional file 10) [34-39]. The RNA-seq data were not derived from strand-specific sequencing. Hence, it was not possible to determine transcription orientation for transcripts that do not contain introns. Maize ESTs including full-length cDNAs used by Boerner and McGinnis [30] from a vast variety of tissues and stages were also collected from GenBank and integrated with the maize B73 genome annotation (AGP v2) (Additional file 10) [32].

### Bioinformatic pipeline for identifying lncRNAs

The different sequence datasets were merged into one non-redundant set of transcript isoforms in maize, which was subjected to a series of filters to eliminate potential protein-coding transcripts (Figure 1).

For the RNA-seq data, all sequenced reads from each experiment were aligned to the maize reference genome (AGP v2) using the spliced read aligner TopHat [33]. Then, a method of two iterations of TopHat alignments proposed by Cabili *et al*. [3] was employed to maximize the use of splice site information derived from all samples. We then re-aligned each experiment using the pooled splice sites file. The transcriptome of each experiment was assembled separately using Cufflinks [40]. To reduce transcriptional noise, only those assembled transcript isoforms that were detected in two or more experiments were retained for further analyses. Then, we compared the assembled transcript isoforms with the maize genome annotation WGS, which represents all transcript isoforms identified by the maize genome project [32]. The RNA-seq dataset enabled us to identify 17,696 transcript isoforms from 16,759 unknown genomic loci after filtering with the WGS. For the maize genome annotation-based transcripts [32], we combined maize ESTs and the WGS to eliminate transcripts from the WGS that were *in silico* annotated without expression evidence. The non-redundant transcripts supported by ESTs and/or RNA-seq were further filtered as follows (Figure 1).

#### Size selection

Putative lncRNAs were arbitrarily defined as transcripts that are ≥200 bp and have no or weak protein coding ability [1-28]. We used in house perl scripts to first exclude transcripts smaller than 200 bp.

#### Open reading frame filter

More than 95% of protein-coding genes have ORFs of more than 100 amino acids [67]. To remove transcripts with long ORFs, which are more likely to encode proteins, a Perl script was developed to ensure that transcripts that encode ORFs of 100 or less amino acids or incomplete ORFs were considered as lncRNA candidates.

#### Known protein domain filter

Transcripts were aligned to the Swiss-Protein database to eliminate transcripts with potential protein-coding ability (cutoff E-value ≤0.001).

#### Protein-coding-score test

The Coding Potential Calculator [41], which is based on the detection of quality, completeness, and sequence similarity of the ORF to proteins in current protein databases, was utilized to detect putative protein encoding transcripts with default parameters. Only transcripts that did not pass the protein-coding-score test were classified as lncRNAs.

#### Elimination of housekeeping lncRNAs and precursors of small RNAs

To rule out housekeeping lncRNAs (including tRNAs, snRNAs, and snoRNAs), putative lncRNAs were aligned to housekeeping lncRNA databases. The housekeeping lncRNA databases include the tRNA database downloaded from the Genomic tRNA Database [68]; the rRNA database from the TIGR Maize Database [69]; and the snRNAs, snoRNAs, and signal recognition particle (7SL/SRP) collected from NONCODE [70]. lncRNA candidates that have significant (*P* < 1.0E-10) alignment with housekeeping lncRNAs were not included in further analyses. Small RNAs in maize, which mainly consist of miRNAs, shRNAs and siRNAs, are generated from their precursors. The small RNA precursors are a special kind of lncRNA. To uncover this kind of lncRNA, we aligned putative lncRNAs with small RNA datasets [71] from multiple tissues, including leaf, ear, tassel, pollen, shoot and root, and different small RNA-related mutants, *mop1* and *rmr2*[72-74], using the same cutoff values used by Boerner and McGinnis [30]. Here, we treated the putative lncRNAs containing homologous sequences to small RNAs as likely precursors of small RNAs; however, some of them may indeed belong to lncRNAs. Conversely, although HC-lncRNAs have no significant alignment with small RNAs, they may still be precursors of small RNAs, which could be expressed at such low levels that they could not be detected using current sequencing technology. Moreover, we also annotated lncRNAs by RepeatMasker [75] (repetitive database version 20130422 from [76]) with default parameters. For the classification of anatomical relationships between lncRNA loci and protein-coding genes, ZmB73 5b annotation [32] was employed to distinguish intergenic lncRNAs from neighboring protein-coding genes. The source code for the lncRNA identification pipeline was released in the GitHub Repository [77].

The above protocol used to identify lncRNAs is similar to previous studies in mammals and plants [3-8,21-30]. However, we employed more stringent criteria than did Boerner and McGinnis [30]. We used ORF ≤100 amino acids as the cutoff, whereas Boerner and McGinnis [30] used <120 amino acids, and double filters of protein-coding potential (known protein domain filter and Protein-coding-score test).

### Validation of putative lncRNAs by RT-PCR

To validate the putative lncRNAs we identified, we conducted RT-PCR of 24 putative lncRNAs in B73 and Mo17 tissues. We grew 10 plants of B73 and Mo17 and sampled the roots, leaves and shoot apices from 14-day-old seedlings. RNA from the roots, leaves and shoots of B73 and shoots of Mo17 was isolated and used for first-strand cDNA reverse transcription by ImProm-IITM Reverse Transcription System (Promega, Madison, WI, USA). A total of 24 putative lncRNAs were randomly selected for validation and RT-PCR was conducted on the lncRNAs using routine PCR programs (Tm = 60°C) with 35 amplification cycles. To control for genomic DNA contamination in our samples, the housekeeping gene *Actin* was used an as experimental control. All primer information can be found in Additional file 11.

### Sequence conservation of FGS, lncRNAs, intergenic and intronic fragments in *Arabidopsis*, rice and sorghum

We employed 1,000 permutations of random sequences for the significance test of sequence conservation of the FGS, lncRNAs, and intergenic and intronic fragments as follows. First, we generated intronic and intergenic annotation files based on the maize WGS annotation [32] and all transcripts identified using the RNA-seq data in this study. Second, we randomly selected a specific number (the same to that of HC-lncRNAs) of intronic and intergenic genomic regions. Third, we adjusted the selected genomic regions according to the transcript length distribution of HC-lncRNAs. Fourth, we obtained the sequences of selected genomic regions based on the repeat-masked reference genome sequence. Fifth, we aligned the randomly selected and length-adjusted intronic and intergenic sequences against the whole genomes of *Arabidopsis*, rice and sorghum. Sixth, we summarized the proportion of aligned selected genomic regions with the *Arabidopsis*, rice and sorghum genomes with a cutoff E ≤1.0E-10. Seventh, we repeated steps 2 to 6 until the total permutation number reached up to 1,000.

### Expression, inheritance and genetic mapping of lncRNAs

Two RNA-seq datasets were collected for the analyses of variation in lncRNA expression among tissues or among different genotypes: 1) RNA-seq data from 13 distinct tissues of the inbred line B73 from 30 experiments [34-39]; and 2) RNA-seq data from 2-week old seedling shoot apices of 105 maize RILs [31].

The expression levels (RPKM) of all transcripts were quantified in the two RNA-seq datasets and normalized using Cufflinks v0.9.3 [40] based on the uniquely mapped reads of each sample. The FGS genes [32], most of which are conserved among species and more likely to be protein-coding genes, were used as controls for the analysis. Tissue-specific analysis measured by Shannon entropy [45] was conducted by expression-level profiling comparison between lncRNAs and the FGS. As previously reported [43], bisulfite sequencing was conducted on the DNA extracted from the third seedling leaf of B73. The DNA methylation levels in CG, CHG and CHH contexts of B73 were calculated for genomic regions from which lncRNAs are transcribed and the FGS and their flanking 1 kb genomic regions [43]. H3K27me3 levels of lncRNAs and FGS were obtained from data reported by Makarevitch *et al*. [46]. For comparison of epigenetic levels, transcription start and stop sites, and upstream and downstream regions were classified based on ZmB73 5b annotations [32].

In our previous study [31], RNA-based sequencing by Illumina Hi-Seq2000 with 103 to 110 cycles were conducted on the pooled RNA samples of 2-week-old seedling shoot apices from three replicates per genotype for 105 maize intermated B73 × Mo17 recombinant inbred lines (IBM-RILs), which were derived from the cross of the inbred lines B73 and Mo17 [78]. Uniquely mapped reads were employed to quantify the expression levels of lncRNAs and the FGS [31]. lncRNAs and genes comprising the FGS, which were detected in the IBM-RILs, were extracted for expression inheritance pattern analysis and genetic mapping. To quantify the expression inheritance of transcripts in the RILs relative to B73 or Mo17, we used a statistic calculated by (Exp_parents_ - μ_progeny_)/σ_progeny_, where Exp_parents_ shows the expression level in the two parents, μ_progeny_ indicates the mean value of the expression level in the progeny population and σ_progeny_ represents the standard variation of the expression level in the progeny population for a specific gene. Any specific transcript could have two adjusted values, which measure the expression level deviation from that of the two parents (Figure 5E). The higher value the statistic is, the more deviation the transcript exhibits in the progeny compared with that of the parents. This statistic is expected to be centered at zero if the RILs generally have expression levels similar to the parents.

A high-resolution SNP genetic map of the IBM population based upon 7,856 high quality SNP markers from RNA-seq data was used to perform eQTL mapping for lncRNAs and FGS by using composite interval mapping. To obtain a global significance of 0.05 for the eQTL mapping, a permutation threshold was computed using 1,000 randomly selected e-traits × 1,000 replicates. This threshold gave a likelihood ratio test value of 19.23, which corresponds to a LOD score of 4.17 as the significant cutoff of eQTL mapping. The confidence interval of eQTL was selected based on the range of a 1.0 LOD drop on each side from the LOD peak point. If two adjacent peaks overlap in less than 10 cM, we considered them as one eQTL [31].

## Abbreviations

bp: base pair; eQTL: expression quantitative trait locus; EST: expressed sequence tag; FGS: filtered gene set; H3K27me3: trimethylation of lysine 27 of histone 3; HC-lncRNA: high confidence lncRNA; IBM-RIL: intermated B73 × Mo17 recombinant inbred line; lncRNA: long noncoding RNA; LOD: logarithm of odds; miRNA: microRNA; ORF: open reading frame; PCR: polymerase chain reaction; RIL: recombinant inbred line; RPKM: reads per kilobase per million reads; SAM: shoot apical meristem; shRNA: short hairpin RNA; siRNA: small interfering RNA; snoRNA: small nucleolar RNA; SNP: single-nucleotide polymorphism; snRNA: small nuclear RNA; WGS: working gene set.

## Competing interests

The authors declare that they have no competing interests.

## Authors’ contributions

LL, NMS, and GJM conceived the project idea. LL, SRE, NMS, JY and GJM performed data analysis. LL, RS, KP, CTY, WW, AMC, SAG, RAC, JEF, MMSE, MJS, PSS, and MCPT performed data collection. JY, PSS, MJS, and MCPT edited the manuscript. LL, NMS and GJM wrote the manuscript. All authors read and approved the final manuscript.

## Supplementary Material

Additional file 1: Table S1 Characteristics of all putative lncRNA identified in this study. Click here for file

Additional file 2: Dataset S1 Annotation of pre-lncRNAs in the format of GTF. Click here for file

Additional file 3: Dataset S2 Annotation of HC-lncRNAs in the format of GTF. Click here for file

Additional file 4: Figure S1 Methylation levels of HC-lncRNAs and FGS genes. Percentage of DNA methylation in CG (black), CHG (red) and CHH (green) contexts is shown for HC-lncRNAs (solid lines) and FGS genes (dashed lines). Dashed vertical lines represent the presumed transcription start (left) and stop (right) for each lncRNA or gene with the length normalized to a value of 1,000. Regions to the left and right of the vertical dashed lines show DNA methylation levels in the 1,000 bp upstream of the presumed transcription start site (based upon ZmB73 5b annotations) or 1,000 bp downstream of the presumed transcription stop site, respectively. Click here for file

Additional file 5: Figure S2 H3K27me3 levels in maize HC-lncRNAs. **(A)** Variation in levels of H3K27me3 in HC-lncRNAs in different tissues of B73. The average level of H3K27me3 was plotted over the gene length (0 to 1,000 represent the normalized length of each HC-lncRNA from presumed transcriptional start to presumed stop while the 1,000 bp upstream or downstream are actual lengths showing the level of H3K27me3 in surrounding regions) for five different tissues. **(B)** H3K27me3 levels of expression and silent HC-lncRNAs in each of the five different tissues. In each tissue, the genes were classified as not expressed (FPKM = 0) or expressed (FPKM >1). Click here for file

Additional file 6: Table S2 eQTL mapping of HC-lncRNA expressed in more than 80% of the RILs. ^a^Chromosome position of e-traits. ^b^Genetic position of e-traits. ^c^The physical chromosomal location on the B73 reference genome (AGPv2) of e-traits. ^d^The middle physical position (equals the sum of the position of the transcription start site and the termination site divided by 2) of e-traits. ^e^The genetic position of the peak of the eQTL. ^f^The genetic position of the inferior support interval left bound of the eQTL. ^g^The genetic position of the inferior support interval right bound of the eQTL. ^h^The physical position of the peak of the eQTL on the B73 reference genome (AGPv2). ^i^The logarithm of odds (LOD) score of the eQTL. ^j^The additive effect - the positive value indicates that the allele from Mo17 increases the phenotypic value. ^k^The amount of expression variation of the e-trait explained by the eQTL. Type shows the relationship between e-traits and the eQTLs. Click here for file

Additional file 7: Table S3 eQTL mapping of HC-lncRNA expressed in more than 40% but less than 80% of the RILs. ^a^Chromosome position of e-traits. ^b^Genetic position of e-traits. ^c^The physical chromosomal location on the B73 reference genome (AGPv2) of e-traits. ^d^The middle physical position (equals the sum of the position of the transcription start site and the termination site divided by 2) of e-traits. ^e^The genetic position of the peak of the eQTL. ^f^The genetic position of the inferior support interval left bound of the eQTL. ^g^The genetic position of the inferior support interval right bound of the eQTL. ^h^The physical position of the peak of the eQTL on the B73 reference genome (AGPv2). ^i^The logarithm of odds (LOD) score of the eQTL. ^j^The additive effect - the positive value indicates that the allele from Mo17 increases the phenotypic value. ^k^The amount of expression variation of the e-trait explained by the eQTL. Type shows the relationship between e-traits and the eQTLs. Click here for file

Additional file 8: Figure S3 The percent of RILs with expressed HC-lncRNAs and population mean of their expression levels in the RILs. The x-axis represents the percentage of RILs, while the y-axis indicates the population mean of RPKM. Click here for file

Additional file 9: Figure S4 LncRNA expression pattern across key stages in embryo development. The y-axis in each panel represents the scaled expression level among key stages (Pro, proembryo; Trans, transition stage; L1, L1 stage; L14, L14 stage; Col, coleoptile stage; and LM, lateral meristem). Each line indicates one gene (in grey) or lncRNA (in blue). The red line shows the mean expression levels in each panel. The title shows the name of the expression level cluster and the number (in brackets) of genes and lncRNAs in each cluster. Click here for file

Additional file 10: Table S4 Datasets used in this study. The preliminary RNA-seq analyses were conducted using TopHat [33] and Cufflinks [40] with the B73 reference genome AGPv2 [32]. Click here for file

Additional file 11: Table S5 Primer information used for lncRNA validation. Click here for file
