# Correction to: Genome-wide discovery and characterization of maize long non-coding RNAs

**DOI:** 10.1186/s13059-018-1508-z

**Published:** 2018-08-23

**Authors:** Lin Li, Steven R. Eichten, Rena Shimizu, Katherine Petsch, Cheng-Ting Yeh, Wei Wu, Antony M. Chettoor, Scott A. Givan, Rex A. Cole, John E. Fowler, Matthew M. S. Evans, Michael J. Scanlon, Jianming Yu, Patrick S. Schnable, Marja C. P. Timmermans, Nathan M. Springer, Gary J. Muehlbauer

**Affiliations:** 1 Department of Agronomy and Plant Genetics, University of Minnesota, Saint Paul, MN 55108 USA; 2 Department of Plant Biology, University of Minnesota, Saint Paul, MN 55108 USA; 3 Department of Plant Biology, Cornell University, Ithaca, NY 14853 USA; 4 Cold Spring Harbor Laboratory, Cold Spring Harbor, NY 11724 USA; 5 Department Agronomy, Iowa State University, Ames, IA 50011 USA; 6 Center for Plant Genomics, Iowa State University, Ames, IA 50011-3650 USA; 7 Current address: Pioneer Hi-Bred, Johnston, IA 50131 USA; 8 Department of Plant Biology, Carnegie Institution for Science, Stanford, CA 94305 USA; 9 Informatics Research Core Facility, University of Missouri, Columbia, MO 65211 USA; 10 Department of Botany and Plant Pathology, Oregon State University, Corvallis, OR 97331 USA

## Correction

The original version [1] of this article unfortunately contained a mistake. The additive effects of the eQTLs of lncRNAs were flipped, meaning that the base allele in the contrast to derive the additive effects should have been B73, rather than Mo17, due to the original coding of biallele SNPs as “0s” and “1s”. Going through the entire analysis procedure, it was determined that the mistake was made while tabulating the eQTL results from QTL Cartographer. The eQTL locations, the proportion of cis- and trans- eQTLs and the eQTL effects for lncRNAs as reported in the original manuscript are all correct. The effect directions of the eQTLs for lncRNAs was not reported. Hence, the correction to reverse the effect directions of the eQTLs is reflected below and in the attached updated Additional file 6 (Table S2) and Additional file 7 (Table S3). The original article was corrected.

In table S2 footnote j and Table S3 footnote j, “the allele from ***Mo17*** increases the phenotypic value” has been corrected to “the allele from ***B73*** increases the phenotypic value”.

The flipped additive effect mistake does not affect other portions of the paper.

## Additional files

Additional file 6: **Table S2.** eQTL mapping of HC-lncRNA expressed in more than 80% of the RILs. a Chromosome position of e-traits. b Genetic position of e-traits. c The physical chromosomal location on the B73 reference genome (AGPv2) of e-traits. d The middle physical position (equals the sum of the position of the transcription start site and the termination site divided by 2) of e-traits. e The genetic position of the peak of the eQTL. f The genetic position of the inferior support interval left Li et al. Genome Biology 2014, 15:R40 Page 12 of 15 http://genomebiology.com/2014/15/2/R40 bound of the eQTL. g The genetic position of the inferior support interval right bound of the eQTL. h The physical position of the peak of the eQTL on the B73 reference genome (AGPv2). i The logarithm of odds (LOD) score of the eQTL. j The additive effect - the positive value indicates that the allele from B73 increases the phenotypic value. k The amount of expression variation of the e-trait explained by the eQTL. Type shows the relationship between e-traits and the eQTLs. (XLS 143 kb)

Additional file 7: **Table S3.** eQTL mapping of HC-lncRNA expressed in more than 40% but less than 80% of the RILs. a Chromosome position of e-traits. b Genetic position of e-traits. c The physical chromosomal location on the B73 reference genome (AGPv2) of e-traits. d The middle physical position (equals the sum of the position of the transcription start site and the termination site divided by 2) of e-traits. e The genetic position of the peak of the eQTL. f The genetic position of the inferior support interval left bound of the eQTL. g The genetic position of the inferior support interval right bound of the eQTL. h The physical position of the peak of the eQTL on the B73 reference genome (AGPv2). i The logarithm of odds (LOD) score of the eQTL. j The additive effect - the positive value indicates that the allele from B73 increases the phenotypic value. k The amount of expression variation of the e-trait explained by the eQTL. Type shows the relationship between e-traits and the eQTLs. (XLS 143 kb)
