# Supplementary figures and images for: Genome-wide discovery and characterization of maize long non-coding RNAs
Source: Genome Biol. 2014 Feb 27;15(2):R40. doi: 10.1186/gb-2014-15-2-r40 (PMC4053991; doi:10.1186/gb-2014-15-2-r40)

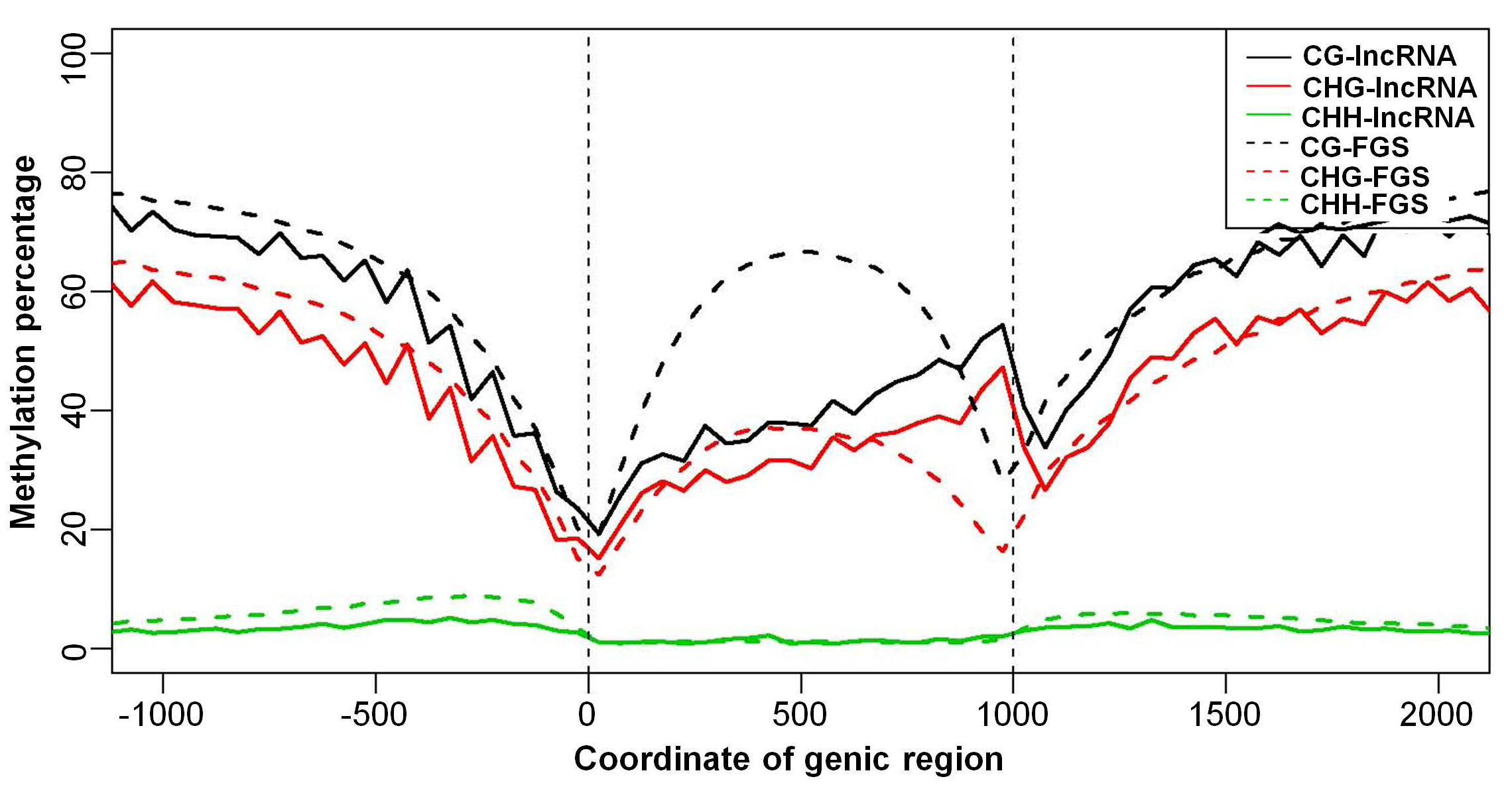

Supplement: Additional file 4: Figure S1 — Methylation levels of HC-lncRNAs and FGS genes. Percentage of DNA methylation in CG (black), CHG (red) and CHH (green) contexts is shown for HC-lncRNAs (solid lines) and FGS genes (dashed lines). Dashed vertical lines represent the presumed transcription start (left) and stop (right) for each lncRNA or gene with the length normalized to a value of 1,000. Regions to the left and right of the vertical dashed lines show DNA methylation levels in the 1,000 bp upstream of the presumed transcription start site (based upon ZmB73 5b annotations) or 1,000 bp downstream of the presumed transcription stop site, respectively. [file gb-2014-15-2-r40-S4.jpeg]

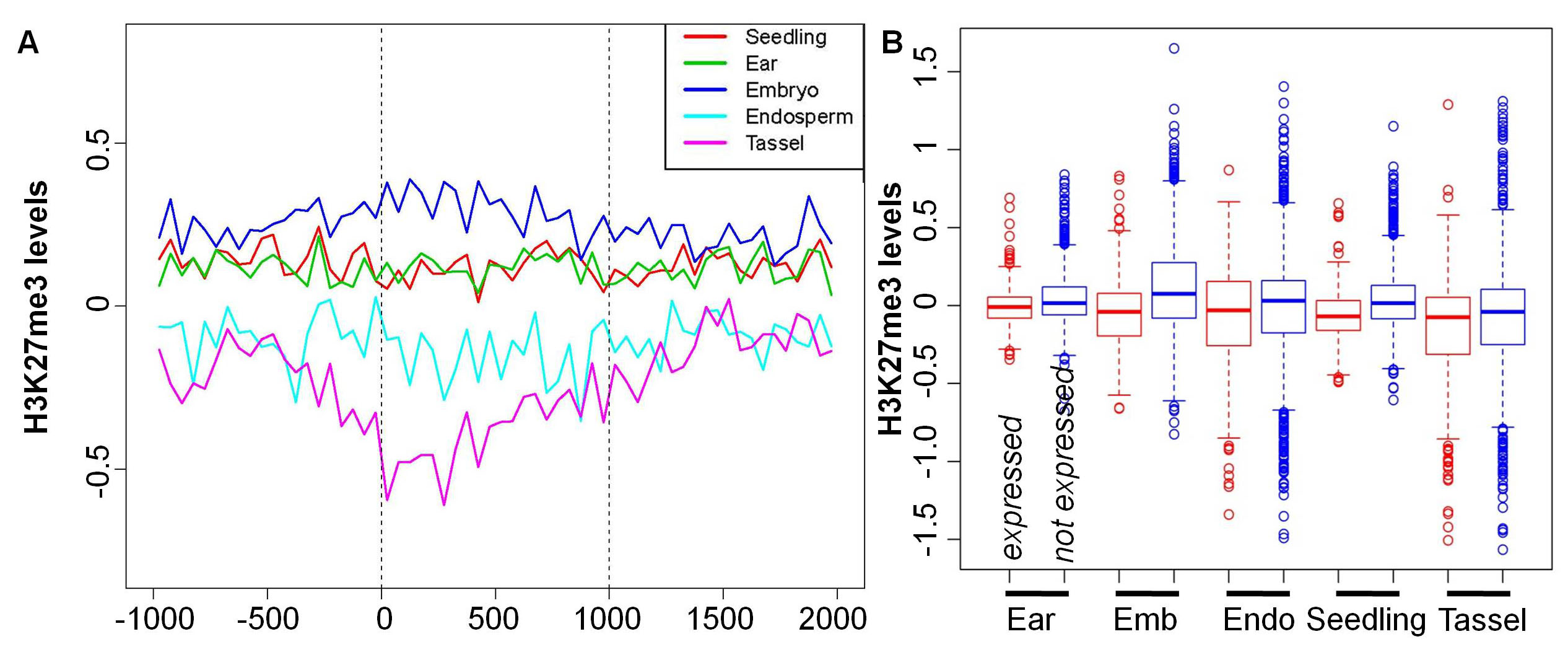

Supplement: Additional file 5: Figure S2 — H3K27me3 levels in maize HC-lncRNAs. (A) Variation in levels of H3K27me3 in HC-lncRNAs in different tissues of B73. The average level of H3K27me3 was plotted over the gene length (0 to 1,000 represent the normalized length of each HC-lncRNA from presumed transcriptional start to presumed stop while the 1,000 bp upstream or downstream are actual lengths showing the level of H3K27me3 in surrounding regions) for five different tissues. (B) H3K27me3 levels of expression and silent HC-lncRNAs in each of the five different tissues. In each tissue, the genes were classified as not expressed (FPKM = 0) or expressed (FPKM >1). [file gb-2014-15-2-r40-S5.jpeg]

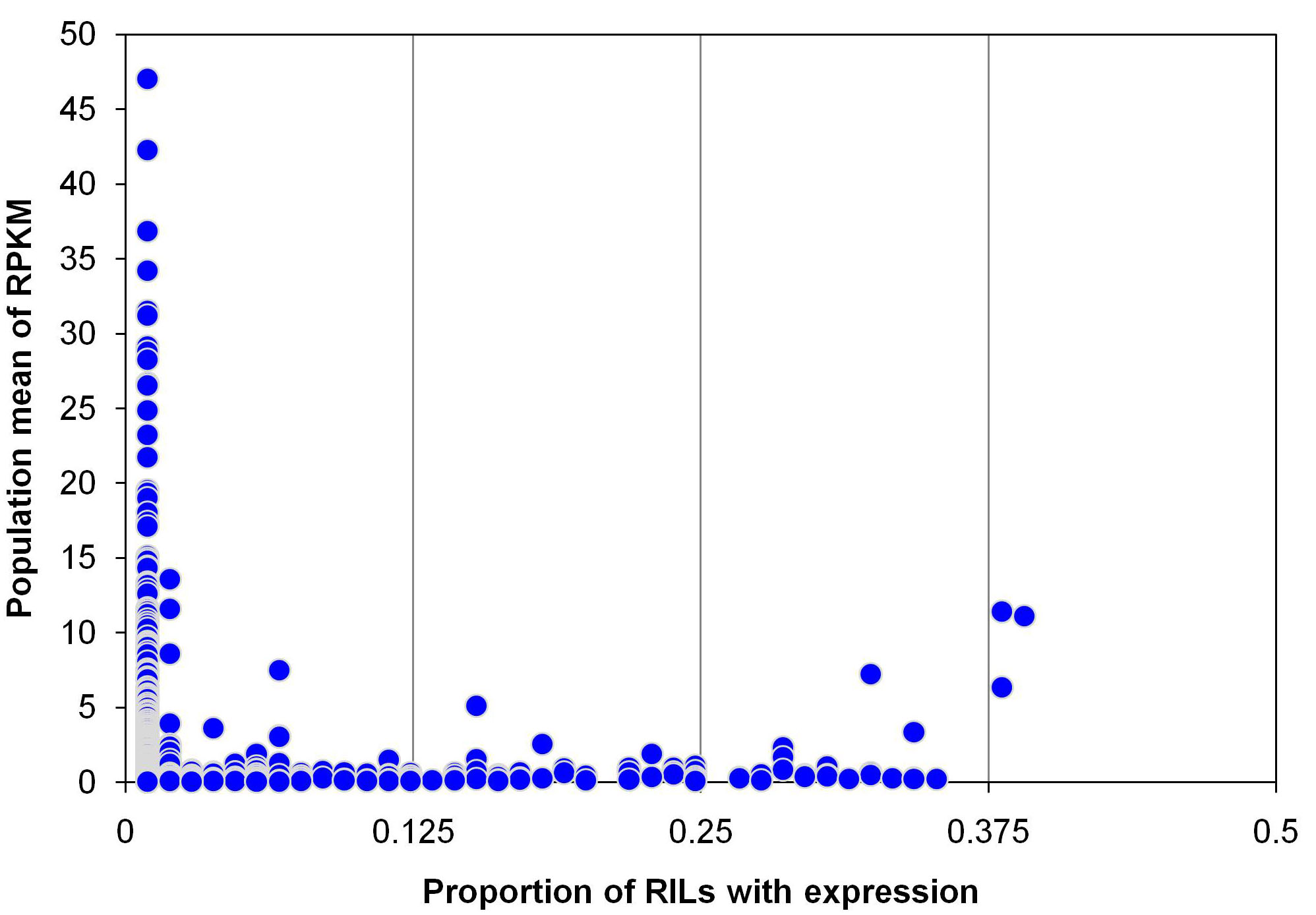

Supplement: Additional file 8: Figure S3 — The percent of RILs with expressed HC-lncRNAs and population mean of their expression levels in the RILs. The x-axis represents the percentage of RILs, while the y-axis indicates the population mean of RPKM. [file gb-2014-15-2-r40-S8.jpeg]

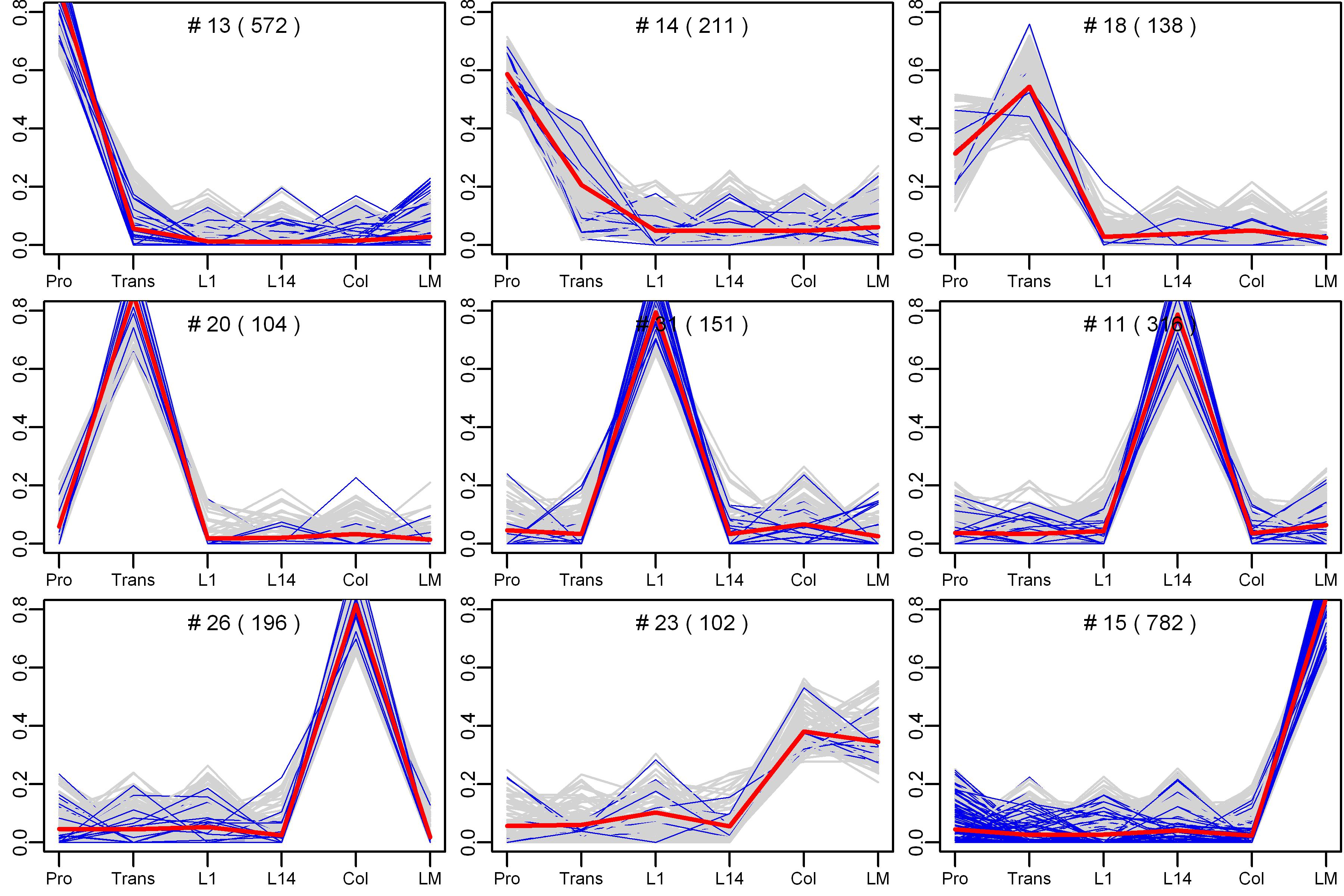

Supplement: Additional file 9: Figure S4 — LncRNA expression pattern across key stages in embryo development. The y-axis in each panel represents the scaled expression level among key stages (Pro, proembryo; Trans, transition stage; L1, L1 stage; L14, L14 stage; Col, coleoptile stage; and LM, lateral meristem). Each line indicates one gene (in grey) or lncRNA (in blue). The red line shows the mean expression levels in each panel. The title shows the name of the expression level cluster and the number (in brackets) of genes and lncRNAs in each cluster. [file gb-2014-15-2-r40-S9.jpeg]
